# An ultra-sensitive multi-channel MEG system for the non-invasive single-trial detection of cortical population spikes

**DOI:** 10.1038/s41598-026-50113-0

**Published:** 2026-05-26

**Authors:** Jim Barnes, Soudabeh Arsalani, Gunnar Waterstraat, Gabriel Curio, Lukasz Radzinski, Jens Haueisen, Rainer Körber

**Affiliations:** 1 Physikalisch-Technische Bundesanstalt (PTB), Abbestrasse 2-12, 10587 Berlin, Germany; 2 Institute of Biomedical Engineering and Informatics, Faculty of Computer Science and Automation, Technische-Universität Ilmenau, 98693 Ilmenau, Germany; 3 Neurophysics Group, Department of Neurology, Charité-Universitätsmedizin Berlin, Corporate Member of Freie Universität Berlin and Humboldt Universität zu Berlin, Hindenburgdamm 30, 12203 Berlin, Germany; 4 Faculty of Electrical Engineering and Computer Science, Technische-Universität Berlin, 10587 Berlin, Germany

**Keywords:** SQUID current sensor, Ultra-low noise dewar, Magnetoencephalography, Somatosensory evoked responses, Engineering, Neuroscience, Physics

## Abstract

We describe the design and performance of a highly sensitive 12-channel magnetoencephalography (MEG) system to be operated in a magnetically shielded room. The aim of the recording system is to measure spatially resolved high frequency (450–850 Hz) somatosensory evoked responses (hfSERs), as elicited by electrostimulation of the median nerve, at a single-trial level. The system uses current sensing superconducting quantum interference devices (SQUIDs) operated in an ultra-low noise dewar. The sensor head consists of 9 multi-turn, hexagonal signal magnetometers, 2 single-turn, circular reference magnetometers and one circular axial gradiometer. Given limitations in the dewar’s size, a balance was found between minimising the magnetic field noise and the spatial distribution of the individual MEG channels. Using SQUIDs currently made at the Physikalisch-Technische Bundesanstalt, the minimum white noise performance is expected to be $\mathrm {\approx 380~aT/Hz^{1/2}}$, with the achieved noise performance in the frequency band of interest of $\mathrm {\approx 500~aT/Hz^{1/2}}$. Additionally, simulations of the neuronal magnetic field of the hfSERs predicted a peak-to-peak strength of 35 fT for our system, in accordance with previous recordings. We also present measures to increase the dynamic range of our MEG device and show in vivo measurement results which demonstrate spatially resolved detection of the hfSERs with amplitudes of $\mathrm {\approx 50~fT_{pp}}$ at the single-trial level.

## Introduction

Superconducting quantum interference devices (SQUIDs) detect magnetic flux with a higher sensitivity than any other device^1^. This sensitivity, along with the ability to record with a temporal resolution of less than 1 ms, led to widespread adaptation in the field of biomagnetism and the development of magnetoencephalography (MEG)^2,3^. In comparison, electroencephalography (EEG), while cheaper, suffers from distortions due to strong influences of the electrical conductivity, in particular the skull, which limits the spatial resolution. Invasive methods, such as electrocorticogram (ECoG)^4^, provide more accurate source localisation^5^, but are limited by a restricted field of view and come with the risk of major (3.4 %, e.g., subdural hematoma) and minor (9.6 %, e.g., bleeding requiring transfusion) complications. In contrast, non-invasive measurements, such as MEG can offer a safer recording method^6^. As invasive recordings require a medical indication, the study of certain diseases such as dementia or depression therefore requires the SQUID’s ability to measure non-invasively at a low-noise level, leaving it well positioned to fill in these knowledge gaps^7^.

Measurements of somatosensory evoked responses (SERs) have been common practice for years in the study of neurology and more specifically neuronal processing^8^. Median nerve stimulation is a long used method for generating these responses^9,10^ and it evokes, in Brodmann area 3b of the brain, the primary low-frequency cortical response (the N20 peak) as well as high frequency SERs (hfSERs , $\mathrm {>450~Hz}$), also known as the $\sigma$-burst^11,12^. High frequency oscillations (HFOs) can also spontaneously occur in the same area of the brain, and they play a useful role in the diagnosis of epilepsy while abnormalities in HFOs are also observed in Parkinson’s disease patients^13,14^. When modelling the source of the high and low frequency SERs as equivalent current dipoles (ECDs), it has been found that their orientation differs^15^. While postsynaptic potentials of pyramidal cells in the somatosensory cortex are considered to be the source of the N20 peak^16^, this work gave rise to the idea that a different population is generating this high frequency burst. However, there has been much debate over what the source is^17–19^.

Until recently, detecting these hfSERs required many hundreds of trials for averaging when using commercial MEG systems with typical noise figures of $\approx$2 fT/Hz$^{1/2}$. An obvious drawback to this method is that a potential variability between trials would be obscured. However, advancements in liquid helium dewar hardware drastically improved SQUID system performance resulting in a noise of roughly $\mathrm{150~aT}/\mathrm{Hz^{1/2}}$^20^. This allowed for the first time the non-invasive single-trial detection of hfSERs using a single-channel MEG system^21^. Analysis of the hfSERs showed that their amplitude and latency are highly variable between trials with an average field strength of up to $\mathrm {\approx 40~fT_{pp}}$ (subject dependent), when detected using a system with a warm-cold distance of 12.9 mm. Additionally, no correlation between the amplitude and latency of the hfSER and the low frequency N20 component could be found. This suggested that hfSERs are not solely controlled by thalamocortical input but separate brain network dynamics.

Optically pumped magnetometers have gained popularity in recent years as an MEG recording method as they do not require liquid helium to function in comparison with SQUIDs, however, their noise performance ($\mathrm{10~fT}/\mathrm{Hz^{1/2}}$) and also limited bandwidth ($\mathrm {<100~Hz}$) prevents them from measuring these hfSERs^22–24^. This demonstrates the clear advantages of SQUIDs in this use case, especially when considering recent improvements made to the system sensitivity. Even though single-channel recordings revealed already an immense amount of information regarding the variability of hfSERs, investigating any potential spatial correlation with neuronal activities in other nearby brain areas, for instance, were impossible and requires simultaneous multi-channel recordings. Hence, in this work, we describe the design, specifications and novel advancements that have been implemented to achieve an ultra-sensitive 12-channel measurement system, built to operate in a moderately magnetically shielded room (MSR). Furthermore, first *in vivo* measurements demonstrate spatially resolved detection of hfSERs at the single-trial level.

## System performance requirements

**Fig. 1:**
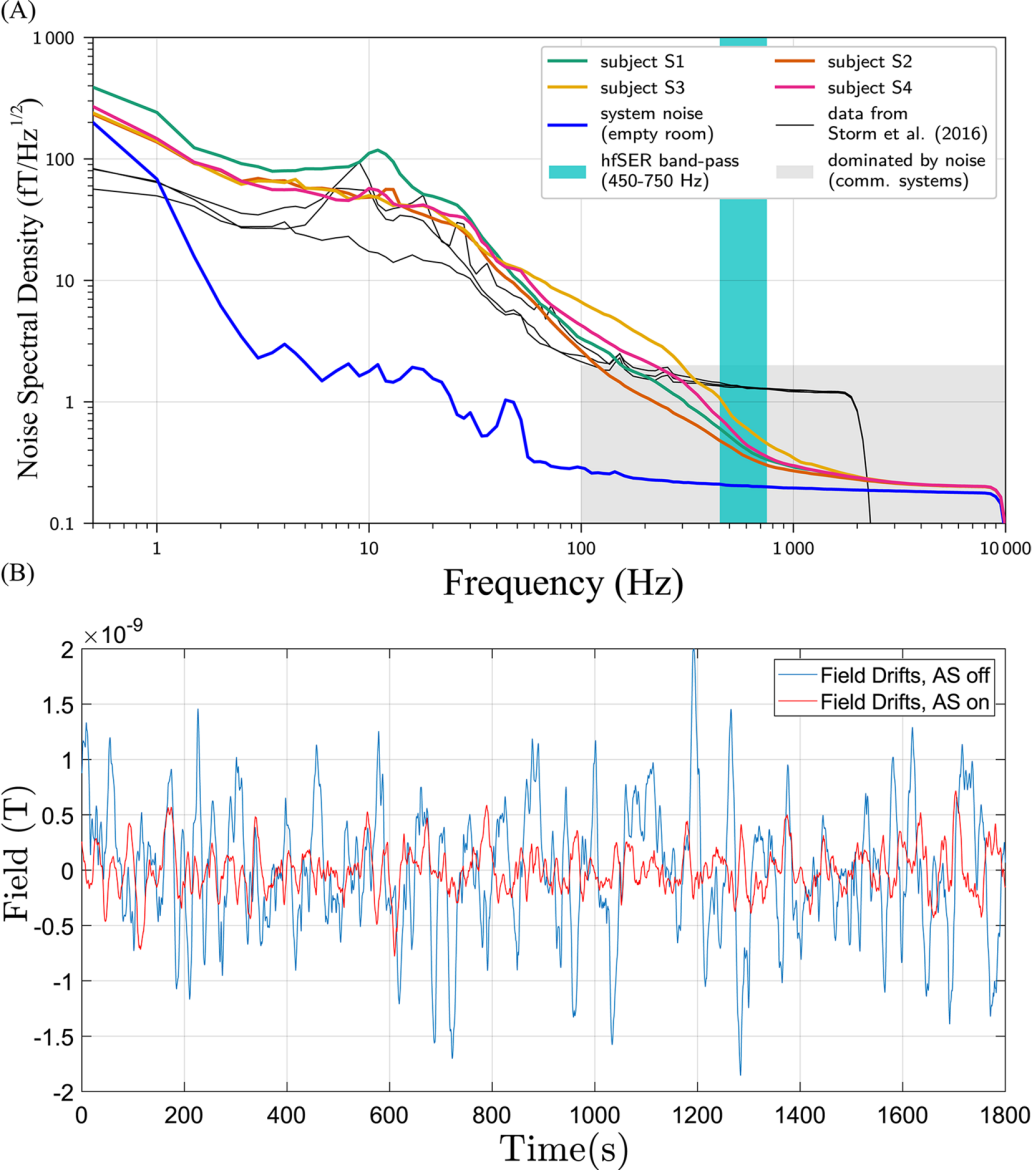
(**A**) Noise spectral density of ultra-low noise SQUID-MEG. One can, for comparison, see recordings with a higher noise system (black lines), the empty room spectrum (dark blue), and the recordings with subjects (the remaining lines), as well as the frequency band of interest, shown in torquoise^21^. (**B**) Field drifts measured in the centre of the ZUSE-MSR with and without active shielding (AS), there is a clear reduction in ranges of recorded field values due to the AS.

A special focus of the design principle of the multi-channel system is the future availability for the general MEG community. Consequently, the usability of the device in a moderately MSR (shielding factor of around 100 at $\mathrm {0.1~Hz}$), such as the Zuse-MSR of the PTB^25^ is the target. The general requirements to be addressed are noise, to detect the spatial pattern of the $\sigma$-burst, and the dynamic range of the entire system.

Starting first with noise issues, magnetic noise from the liquid helium dewar can be eliminated by constructional measures^26^. Based on our previous work^20^ we have upgraded another commercial low noise dewar (Cryoton LH-16.4 NTE) to have negligible noise. The dewar has a flat bottom finger with an available diameter of 70 mm for the sensor head. Next, the mean measured field noise in the centre of the Zuse-MSR amounts to $\approx$325 aT/Hz$^{1/2}$ in the $\sigma$-burst range (450-850 Hz) which can be suppressed by gradiometry. In Fig. 1A, the noise spectral density measured with an ultra-sensitive first-order gradiometer of 45 mm diameter and 120 mm baseline is shown (blue curve). The white noise performance is $\mathrm {\approx 180~aT/Hz^{1/2}}$ and close to intrinsic SQUID noise performance could be achieved, enabling the reliable single-trial detection of hfSERs^21^. Remaining physiological activity in the $\sigma$-band, indicated by the torquoise band, depends on the subject and is on average $\mathrm {\approx 430~aT/Hz^{1/2}}$. The novel multi-channel system should be capable of differentiating between this and environmental noise contributions. Consequently, due to field fluctuations in the MSR, gradiometry is required. For a given loop size, a magnetometer has a lower intrinsic noise (ideally $\sqrt{2}$ times smaller) compared to standard hardware gradiometer (expressed as noise referred to the pick-up loop). Therefore, we opt for separate reference magnetometer channels which can be used for synthetic gradiometry in a postprocessing step. As will be shown below, the reference magnetometers can be made to have minimal noise compared to the signal channels. In addition, software gradiometry allows adjustment of the processing parameters.

Increasing the pick-up coil area does improve the field noise, but the multi-channel detection of the hfSERs requires a balance between sufficiently large pick-up loops, able to detect the minute field changes at the femtotesla level, but small enough that they adhere to the physical limitations of the ultra-low noise dewar, while also being able to sample the field distribution of the hfSERs. Considering the whole SQUID system, the noise of the analog-to-digital converter used for data acquisition must also be considered. It is desirable that the SQUID circuit produces the dominant noise contribution and is the limiting factor in performance of the overall system. In accounting for the body noise in the $\sigma$-burst range, based on the distance from the pick-up loop to our subject as well as our pick-up loop radii, we can estimate this to be to be $\mathrm {\approx 60~aT/Hz^{1/2}}$^27^. Once the field noise reaches values of $\mathrm {\approx 380~aT/Hz^{1/2}}$ this contribution is $\approx 1\%$, so we can assume it does not play a critical role.

Apart from noise considerations, the operation in a moderately MSR, as opposed to a heavily shielded environment like the BMSR2.1 of PTB (shielding factor of above $10^6$ at $\mathrm {0.1~Hz}$), imposes further challenges concerning the dynamic range of the entire SQUID system. Alongside magnetic fields from the brain, field drifts will also occur inside the MSR. Low-frequency disturbances are primarily observed in the vertical *z*-direction and are caused by the Berlin underground nearby. Active shielding (AS) is in place which consists of compensation coils installed outside the MSR and a fluxgate in a negative feedback circuit^25^. Figure 1B shows the field drifts that occur in the MSR in a 30 minute recording session with and without AS. The drifts in the MSR are suppressed by the AS approximately 2.5 times (3.85 nT down to 1.49 nT). These drifts need to be recorded by the signal channels and the more sensitive reference channels with sufficient dynamic range so that overall system performance is not compromised. 

## Design of the 12-channel SQUID system

**Fig. 2:**
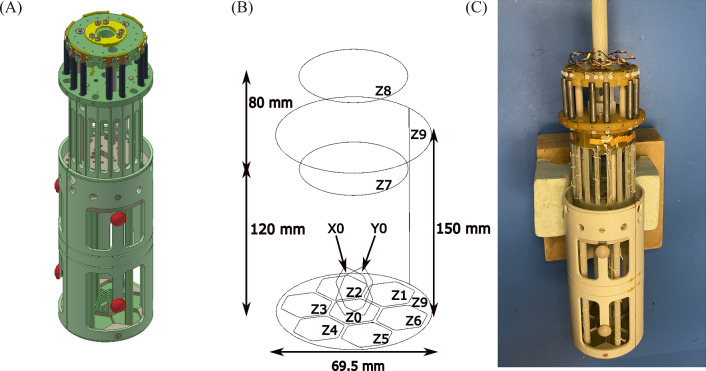
Sensor probe designs. (**A**) A CAD rendering of the sensor probe. Green material is GFRP while the beige material is PEEK. The red pieces are to ensure the sensor probe is centrally located within the dewar. (**B**) Layout of the sensor channels, showing the 9 hexagonal signal channels (Z0–Z6, X0 and Y0), the two larger reference channels (Z7 and Z8) and the first-order axial gradiometer (Z9). (**C**) Photo of the sensor probe complete with wound input coil circuits. Yellow kapton tape is used to fix the wires in place.

The layout of our 12-channel sensor probe consists of 9 signal channels, 2 reference magnetometers and one larger hardware gradiometer to mimic a more sensitive version of the previous single-channel system,  it is shown in Fig. 2A as a CAD rendering and an actual photo is shown in Fig. 2C, with Fig. 2B showing the general sensor layout of the system. Seven hexagonal loops $\mathrm{\ Z0{-}Z6}$ form a closed packed arrangement and are sensitive in the *z*-direction. Neuronal field simulations shown later indicate this design enables spatial coverage of the $\sigma$-burst. A centre-to-centre distance between neighbouring pick-up loops of 21 mm allows for the requisite spatial resolution, while adhering to size limitations. Additionally, a central doublet, sensitive in the *x*- and *y*-direction is positioned with its centre point 10.25 mm above the bottom pick-up loops. Two circular reference magnetometers (Z7 and Z8) with radius 24 mm are located 120 mm and 200 mm above the sensors respectively. The choice of hexagonal signal channels will be explained in the following sections along with details on their field sensitivity as well as that of the reference channels. A first-order axial gradiometer (Z9) with a diameter of 69.5 mm and a baseline of 150 mm surrounds the magnetometers and is designed to be an upgrade on the previous 45 mm diameter gradiometer from the single-channel system^20^. The pick-up coils are connected to their respective SQUIDs via a detachable contact. Each is situated inside its own open ended superconducting niobium cylinders for shielding analogous to the design described by Storm *et al.*^28^. The SQUIDs are read out with the XXF electronics^29^ which can also be situated outside the MSR. The probe itself is mostly made out of polyether ether ketone (PEEK) and glass fibre reinforced plastic (GFRP). Both materials are relatively easy to machine, are electrically insulating, non-magnetic and exhibit a high level of thermal stability.

## SQUID sensor configuration and optimization

**Fig. 3:**
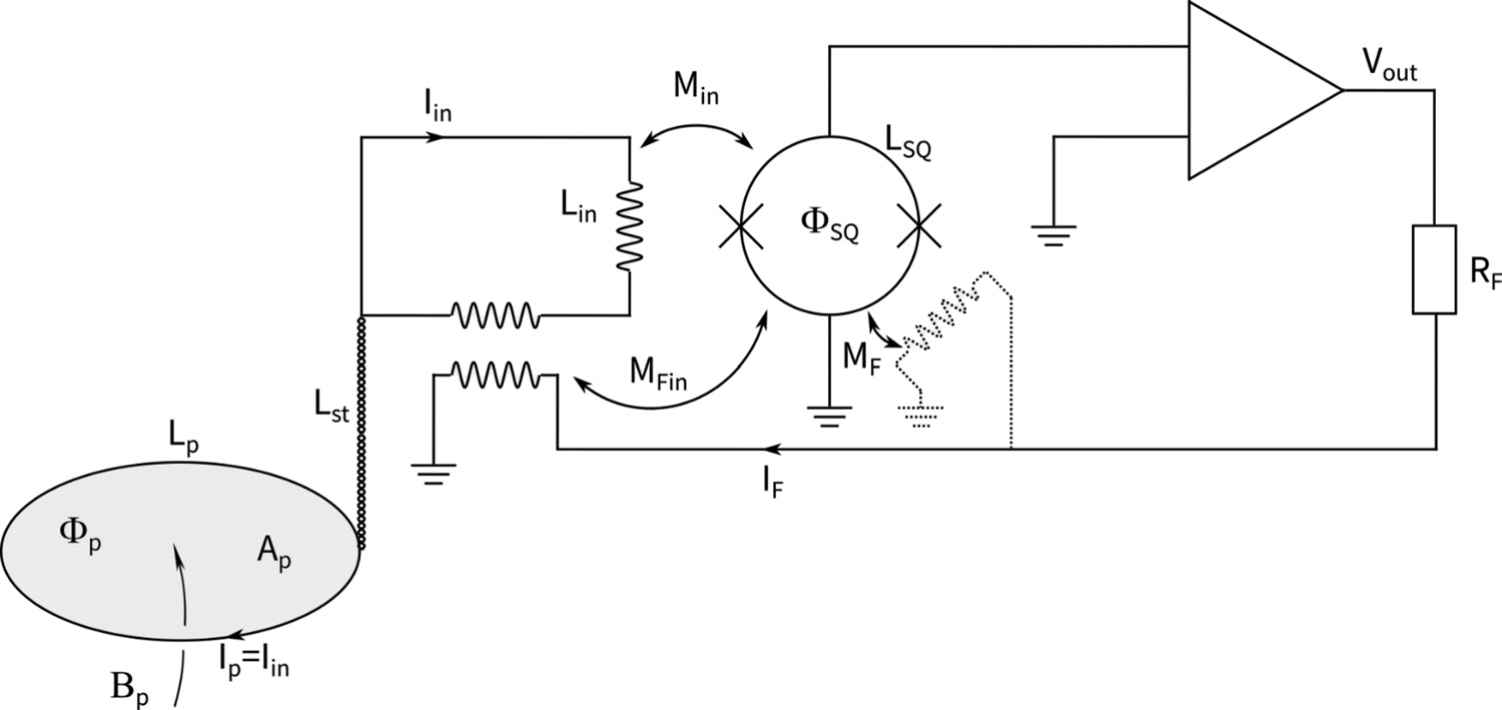
SQUID schematic. On the left side the input coil system can be seen, where a field, $\mathrm {B_p}$, through the pick-up loop induces a current $l_{\textrm{in}}$ that is carried to $\mathrm {L_{in}}$. This creates a flux $(\Phi _{SQ})$ which causes a change in SQUID voltage. The output voltage ($\mathrm {V_{out}}$) is recorded by an ADC while the feedback circuit keeps the magnetic flux through the SQUID constant.

In modelling the sensitivity of our device to changes in magnetic fields, we first consider the noise performance of the SQUID sensor, i.e., the current sensing SQUID coupled to a pick-up coil. As mentioned, magnetic noise from the MSR will be minimized by software gradiometry while dewar and body noise contributions are negligible. Additional noise sources in the signal acquisition chain are treated separately.

A schematic circuit diagram is shown in Fig. 3. For the current sensing SQUID scheme, the appropriate figure of merit is the coupled energy sensitivity per unit bandwidth $\epsilon _{\textrm{c}}=L_{\textrm{in}}S_{\mathrm {\Phi }}/(2M_{\textrm{in}}^{2})$, where $L_{\textrm{in}}$ is the input coil inductance, $S_{\mathrm {\Phi }}$ is the noise flux power per unit bandwidth of the SQUID and $M_{\textrm{in}}$ is the mutual inductance between the SQUID and the input coil. $\epsilon _{\textrm{c}}$ allows the comparison between different current sensing SQUIDs as it takes both the flux noise $S_{\mathrm {\Phi }}$ and the coupling to the input coil into account.

The field sensitivity $B_{\mathrm {\Phi }}=L_{\textrm{tot}}/(M_{\textrm{in}}A_{\textrm{p}})$ gives the required (homogeneous) magnetic field in the pick-up coil of inductance $L_{\textrm{p}}$ and area $A_{\textrm{p}}$ which produces 1 $\Phi _{0}$ in the SQUID. Here, the total inductance of the input circuit $L_{\textrm{tot}}$ is given by $L_{\textrm{tot}}=L_{\textrm{p}}+L_{\textrm{in}}+L_{\textrm{st}}$, where $L_{\textrm{st}}$ denotes any stray inductance due to the twisted pair connecting the pick-up coil to the SQUID input coil. If the pick-up coil consists of a superconducting circular loop, its inductance $L_{\textrm{p}}$ is given by $L_{\textrm{p}}=\mu _{0}r_{\textrm{p}}\left[ \ln \left( 8r_{\textrm{p}}/r_{\textrm{w}}\right) -2\right]$ where $\mu _{0}$ is the permeability of free space, $\mathrm {r_p}$ is the radius of the loop and $\mathrm {r_w}$ is the radius of the wire^30^. For a non-circular coil, $L_{\textrm{p}}$ can be approximated by the inductance of a circular loop enclosing the same area.

Combining the field sensitivity and the flux noise results in the field noise, $S_B^{1/2}$, for the SQUID sensor:

1 $$\begin{aligned} S_{\textrm{B}}^{1/2}=B_{\mathrm {\Phi }}S_{\mathrm {\Phi }}^{1/2}=\frac{L_{\textrm{tot}}}{A_{\textrm{p}}}\left( \frac{2 \varepsilon _{\textrm{c}}}{L_{\textrm{in}}}\right) ^{1/2} \end{aligned}$$

**Fig. 4:**
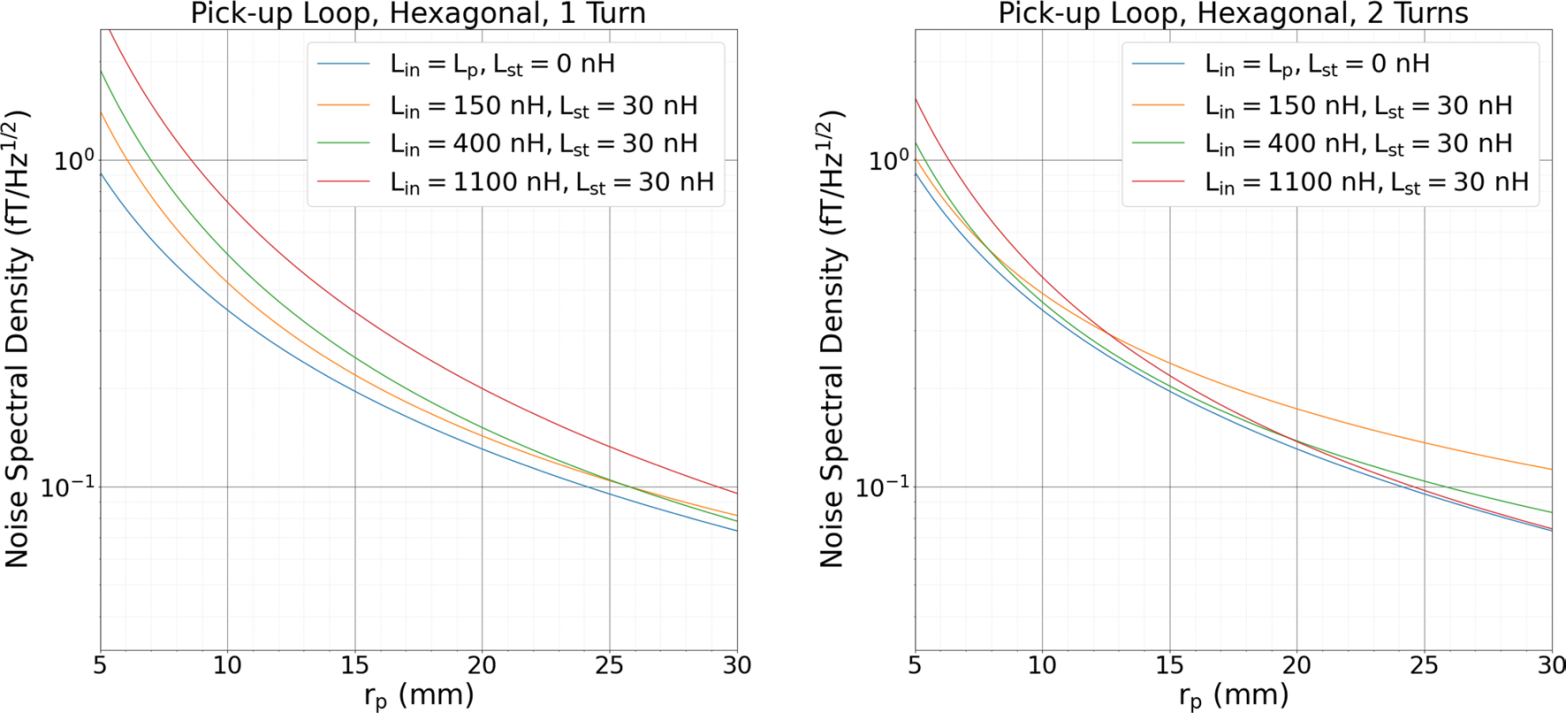
(**A**) Noise spectral density for single-turn hexagonal loop. (**B**) Noise spectral density for two-turn hexagonal loop. For the best case scenario ($\mathrm {L_i=L_p}$ and $\mathrm {L_{st}=0}$) the noise is unchanged, however an improvement is observed when a $\mathrm {L_{st}}$ is accounted for each $\mathrm {L_{in}}$ within certain $r_{\textrm{p}}$ ranges.

While it is flux that a SQUID measures, its output is a voltage. The voltage noise $S_{\textrm{V}}^{1/2}$ can be altered by changing the feedback resistance $R_{\textrm{f}}$, and for a given SQUID sensor, $S_{\textrm{V}}^{1/2}$ is given by $S_{\mathrm {\Phi }}^{1/2}G_{\textrm{FLL}}=S_{\Phi }^{1/2}R_{\textrm{f}}/M_{\textrm{fin}}$. Here, $G_{\textrm{FLL}}$ is the gain in FLL-mode (in units of V/$\Phi _{0}$) and the mutual feedback inductance, $\mathrm{ M_{fin}}$, can be determined using the methods found in Storm et al.^38^. From $B_{\mathrm {\Phi }}$, we can calculate the conversion factor $V_{\textrm{out}}/B=G_{\textrm{FLL}}/B_{\mathrm {\Phi }}=R_\textrm{f}/(M_{\textrm{fin}}B_{\mathrm {\Phi }})$.

In order to determine the optimum layout of our system, noise calculations (Equation 1) in dependence of $r_{\textrm{p}}$ were carried out for the ideal case, i.e. perfect matching ($L_{\textrm{p}}=L_{\textrm{in}}$) and $L_{\textrm{st}}=0$ and for realistic scenarios. Here, parameters of the present PTB current sensor SQUIDs with $\mathrm {L_{in}}$ values of 150, 400 and 1100 nH were considered, which feature a typical $\varepsilon _{\textrm{c}}$ of 37.5 *h*, where *h* is Planck’s constant, and $L_{\textrm{st}}$ was 30 nH. Circular and hexagonal pick-up coils were examined where the latter was parametrized by the inscribed circle of radius $r_{\textrm{p}}$. Compared to a circular coil, a hexagonal coil results in a $\mathrm 6/(\sqrt{3}\pi )$-times larger $A_{\textrm{p}}$ for a given $r_{\textrm{p}}$. Size constraints limited $r_{\textrm{p}}$ to 10.5 mm for circular and 10.25 mm for hexagonal loops, respectively.

**Table 1:** Calculated $S_{\textrm{B}}^{1/2}$ for signal channel and reference channel magnetometers. The signal channels are hexagonal coils whereas the reference channels consist of circular coils. *N* gives the number of turns.

	$S_B^{1/2}$ (aT/Hz$^{1/2}$)			
$L_{\textrm{in}}$ (nH)	Signal channel$^\textrm{a}$		Reference channel$^\textrm{b}$	
	$N=1$	$N=2$	$N=1$	$N=2$
$L_{\textrm{in}}=L_{\textrm{p}}$	332	332	107	107
150	401	376	117	149
400	488	351	120	115
1100	703	416	155	111

$^\textrm{a}$ Hexagonal loop, $r_{\textrm{p}}=10.25$ mm

$^\textrm{b}$ Circular loop, $r_{\textrm{p}}=24$ mm

Figure 4 shows $S_{\textrm{B}}^{1/2}$ of single and double-turn hexagonal pick-up coils. The results for the circular coils are not shown here (the larger $r_{\textrm{p}}$ values give a rough indication of noise performance for the larger circular reference magnetometers) as they are functionally the same with an increased noise for a given $r_{\textrm{p}}$, supporting our choice of hexagonal pick-up coils. For the ideal case, no change in $S_{\textrm{B}}^{1/2}$ is observed when adding an extra turn. However, for the realistic scenario, adding an extra turn to the pick-up loop improves $S_{\textrm{B}}^{1/2}$ within certain $r_{\textrm{p}}$ ranges, as it diminishes the negative effects of $\mathrm L_{\textrm{st}}$. For $L_{\textrm{in}}=400$ nH, $S_{\textrm{B}}^{1/2}$ improves with a second turn when $r_{\textrm{p}}<27$ mm. In addition, Fig. 4B shows, that these parameters results in a performance close to the ideal case over a wide range of $r_{\textrm{p}}$, from about 8 to 22 mm. For $r_{\textrm{p}}<8$ mm, a SQUID with $L_{\textrm{in}}=150$ nH is preferable whereas for $r_{\textrm{p}}>22$ mm, $L_{\textrm{in}}=1100$ nH gives the lowest $S_{\textrm{B}}^{1/2}$. Table 1 shows the expected field noises for each of our different channels. For the signal channels, two turns with a $L_{\textrm{in}}$ of 400 nH give the best noise performance, $\mathrm{ 351~aT/Hz^{1/2}}$. This is only slightly increased to $\mathrm{ 375~aT/Hz^{1/2}}$ when performing software gradiometry using a single-turn reference magnetometer coupled to a current sensing SQUID with $L_{\textrm{in}}=150$ nH. Adding a third turn would result in improved noise performances for a $L_{\textrm{in}}$ of 400 nH, but only when $r_{\textrm{p}}<10$ mm.

## Neuronal field simulations

To consider the expected spatial pattern of the $\sigma$-burst, simulations were performed in MNE-Python^31^. Our model takes into account the size of the pick-up loops, the strength, location and orientation of the ECD for the burst, as well as the distance between our pick-up loops and ECD. This approach allows the prediction of the expected neuronal fields of all pick-up loops.

As the Brodmann area 3b, which is located in the Parietal lobe of the brain^32^, gives a similar response in modelling for spherical or generic MRI models^33^, a spherical model was used. A schematic of the computational model is shown in Fig. 4A. Here, the radii of the spheres enclosed by the brain, cerebrospinal fluid (CSF), skull and scalp are 8.8 cm, 9.0 cm, 9.5 cm and 9.8 cm, while their conductivities are taken to be 0.33 S/m, 1.0 S/m, 0.004 S/m and 0.33 S/m, respectively^31^. Note, a single brain-layer model would also suffice as MEG is insensitive to radial conductivity differences, in this case however, the multilayer model can be readily used for future simulations of EEG signals, for which the conductivity profile matters. To calculate the magnetic field detected by our sensor head, a grid with a spacing of 0.1 mm was created. The field measured in each signal channel was calculated as the effective field within the pick-up loop.

**Fig. 5:**
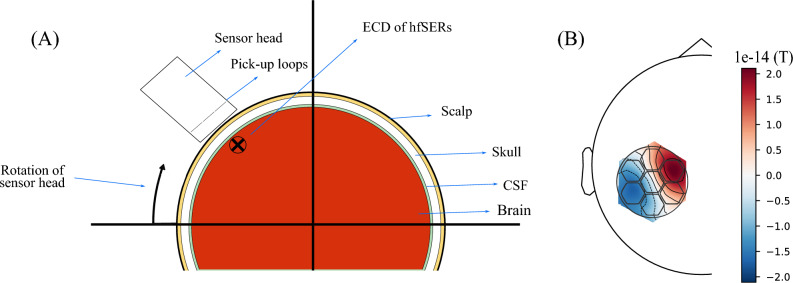
(**A**) Four-layer sphere model. The position of the sensor head in relation to the ECD is when the largest range of field values is detected. (**B**) Neuronal field map for sphere model. The signal channels can detect positive and negative peak values caused by the ECD. The outline of our sensor head is superimposed onto the field showing the size of our device compared to the measured field.

The location of the high frequency burst has been determined during simultaneous EEG-fMRI recordings^34^, while the ECD orientation and strength ($1.6 \pm 0.9~\textrm{nAm}$) has been found through MEG measurements^15^. The dewar warm-cold distance is 14 mm with a distance from the closest pick-up loops to the ECD of 46 mm. Size constraints mean our pick-up loops are enclosed within a circular boundary 66 mm in diameter. A hexagonal arrangement of integrated SQUID magnetometers is common for close-packing of sensors^35,36^, while hexagonal-shaped pick-up loops have also been used before^28,37^. To compare circular versus hexagonal loops in terms of flux detected, an isotropic grid with a discretization of $0.1~\textrm{mm}$ was created. The magnetic field at each grid point was calculated and the flux numerically integrated. The hexagonal coils have an area roughly 6% greater than the circular ones. Simulations were also performed to determine the expected field strength from the ECD at distances where the larger reference magnetometers are located.

In Fig. 5B, the topographical field map for the ideal sensor head position is shown. The size of the sensor head covers a large portion of the dipolar field pattern. A maximum peak-to-peak value in the dipolar field pattern of $\mathrm {35~fT}$ is observed between our pick-up loops. It should be noted that the rotation of the sensor head about its vertical axis can change this result by ca. 10 %. The difference in field detected by hexagonal versus circular pick-up coils was found to be around 5 %, which is commensurate with the increase in area. The reference sensors, Z7 and Z8, detect a relative field strength of the $\sigma$-burst of 1.2 and 0.17 % respectively, when compared to the bottom z-sensors. Thus, software gradiometry will have negligible attenuation of the $\sigma$-burst signal. We also compared this four-layer spherical model with a three-layer BEM model based on the default MRI model MNE-Python offers. The BEM model showed a peak-to-peak value of $\mathrm {34.3~fT}$, showing good agreement and demonstrating the sufficient accuracy of the spherical model for our purpose.

## Noise and dynamic range of the recording system

Knowledge of the field noise of the SQUID sensors and the expected field from the hfSERs is critical, but as mentioned the entire measurement chain must be taken into account when quantifying the overall system performance. It is desirable to have as high an output voltage as possible for the smallest change in flux so the SQUID noise dominates the noise of the measurement system. Our data acquisition consists of a 24-bit digitizer (NI, PXI 4495) which has a measured input referred voltage noise of $\mathrm{ 100~nV/Hz^{1/2}}$ for a $\pm 10~\textrm{V}$ voltage range corresponding to an effective resolution of 20.9 bit.

While noise considerations favour as large an $R_{\textrm{f}}$ as possible, the conversion factor needs to be chosen so that the output voltage of the maximum field change stays within the ±10 V range of the SQUID electronics. This sets an upper bound for $R_{\textrm{f}}$. In the ideal case, $R_{\textrm{f}}$ is chosen such that $S_{\textrm{V}}^{1/2}$ of the SQUID dominates and the maximum expected signal can be detected without saturation. To give an example, consider a hexagonal signal channel with $r_{\textrm{p}}=10.25$ mm, $S_{\mathrm {\Phi }}^{1/2}=0.7~\mu \Phi _0/\text {Hz}^{1/2}$ and $R_{\textrm{f}}=100$ k$\Omega$ connected to a SQUID with $L_{\textrm{in}}=400$ nH. This results in $S_{\textrm{V}}^{1/2}=4.04~\mu$V/Hz$^{1/2}$, roughly 40 times larger than the noise of the ADC. The conversion factor $V_{\textrm{out}}/B$ amounts to $\mathrm{ 11.3~V/nT}$ corresponding to a field range of 1.74 nT before saturation occurs. Hence, the active shielding of the moderate MSR enables recording with a higher $\mathrm R_{\textrm{f}}$ (field range of $\mathrm {1.49~nT}$ with AS compared to $\mathrm {3.85~nT}$ without AS). However, even with active shielding, the reference coils with a $V_{\textrm{out}}/B$ of $\mathrm {44.74~V/nT}$ will lead to saturation for an $\mathrm {R_{f}}$ of $\mathrm{ 100~k\Omega}$.

A method to increase the sensitivity of the system is resetting the SQUID via an external reset pulse. This involves taking advantage of the flux locked loop (FLL) fast switching time, to quickly turn off and on the SQUID. This resets the output voltage if it is outside the voltage caused by $\mathrm \pm \frac{\Phi _0}{2}$ to within that range, reducing the likelihood of saturation. While increasing the dynamic range by flux counting (due to the periodic flux-voltage characteristics) has been done before^38^, our operation relies on resetting the SQUID system at a set time interval. For demonstrating this method, we used a single channel, 22.5 mm radius magnetometer SQUID system operated in the MSR without AS. $\mathrm {R_{f}}$ was set to $\mathrm{ 100~k\Omega}$, the reset pulse had a frequency of $\mathrm {1~Hz}$ and a pulse width of $\mathrm {1~ms}$. For the pulse width period where the SQUID is turned off, the change in the field can be assumed to be negligible.

**Fig. 6:**
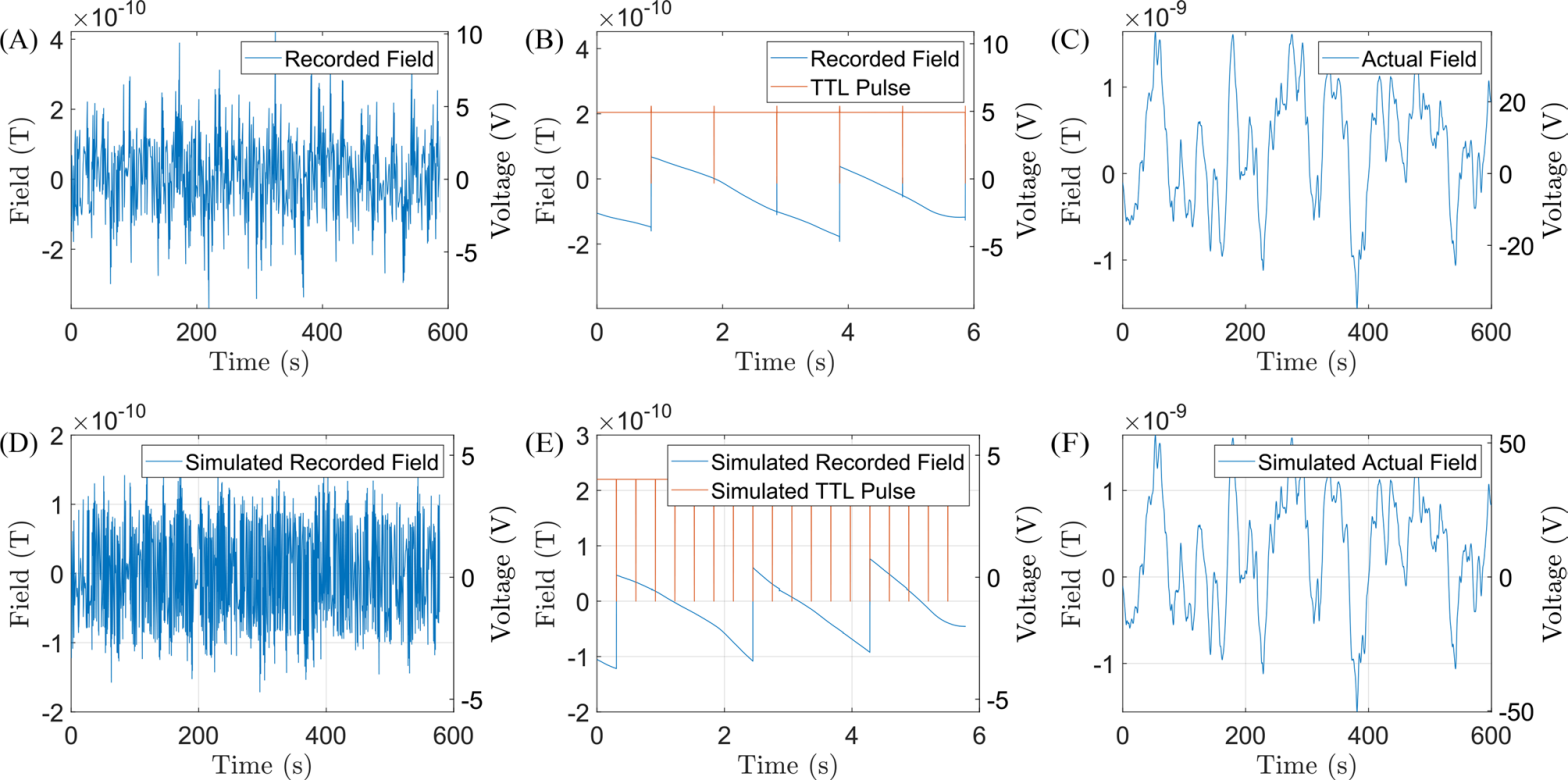
Demonstration of increasing the dynamic range of a SQUID system by utilizing externally activated SQUID reset. (**A**) Recorded field drifts alongside recorded output voltage, a short reset pulse of 1 ms length was applied every second. (**B**) Zoomed in pulse reset, the pulse as well as outputted field/voltage being reset can be seen. (**C)** Actual field drifts after output signal has been pieced together. (**D**) Simulated output voltages of the reference magnetometer for $\mathrm{ {R_{f}}=\mathrm 100~k\Omega}$ when implementing a 3.27 Hz reset pulse. (**E**) Zoomed in reset pulse showing voltage resetting, only when outside $\mathrm \pm \frac{\Phi _0}{2}$ range, (**F**) Theoretical output voltages if the reset pulse was not implemented. Data is functionally equivalent to (**C**).

The results from Fig. 6A, where the SQUID reset is implemented each second, show a field range of roughly $\mathrm{ 0.85~nT}$ for a 10 minute period. However, Fig. 6C shows the actual field drifts with a range of $\mathrm{ 3.25~ nT}$. The pieced together output voltage shows an effective ADC voltage range of $\pm 40 \textrm{V}$. Through this resetting, the dynamic range is increased approximately 4 fold and the overall system performance is less affected by the noise of other components in the system.

One can also project the output voltages for the reference sensors which, due to their higher gain, are more prone to saturation. However, a higher reset frequency of 3.27 Hz, synchronous to the median nerve stimulation, could be used to minimize $\mathrm {V_{out}}$ for a given $\mathrm {R_{f}}$ as shown in Fig. 6D-F. The voltage range is effectively 5 times higher than $\mathrm{ \pm 10~V}$. This procedure can greatly improve system performance when it is hindered by various factors, such as high magnetic field drifts in the MSR or ADCs with higher intrinsic noise.

## System performance and in vivo measurement

**Fig. 7:**
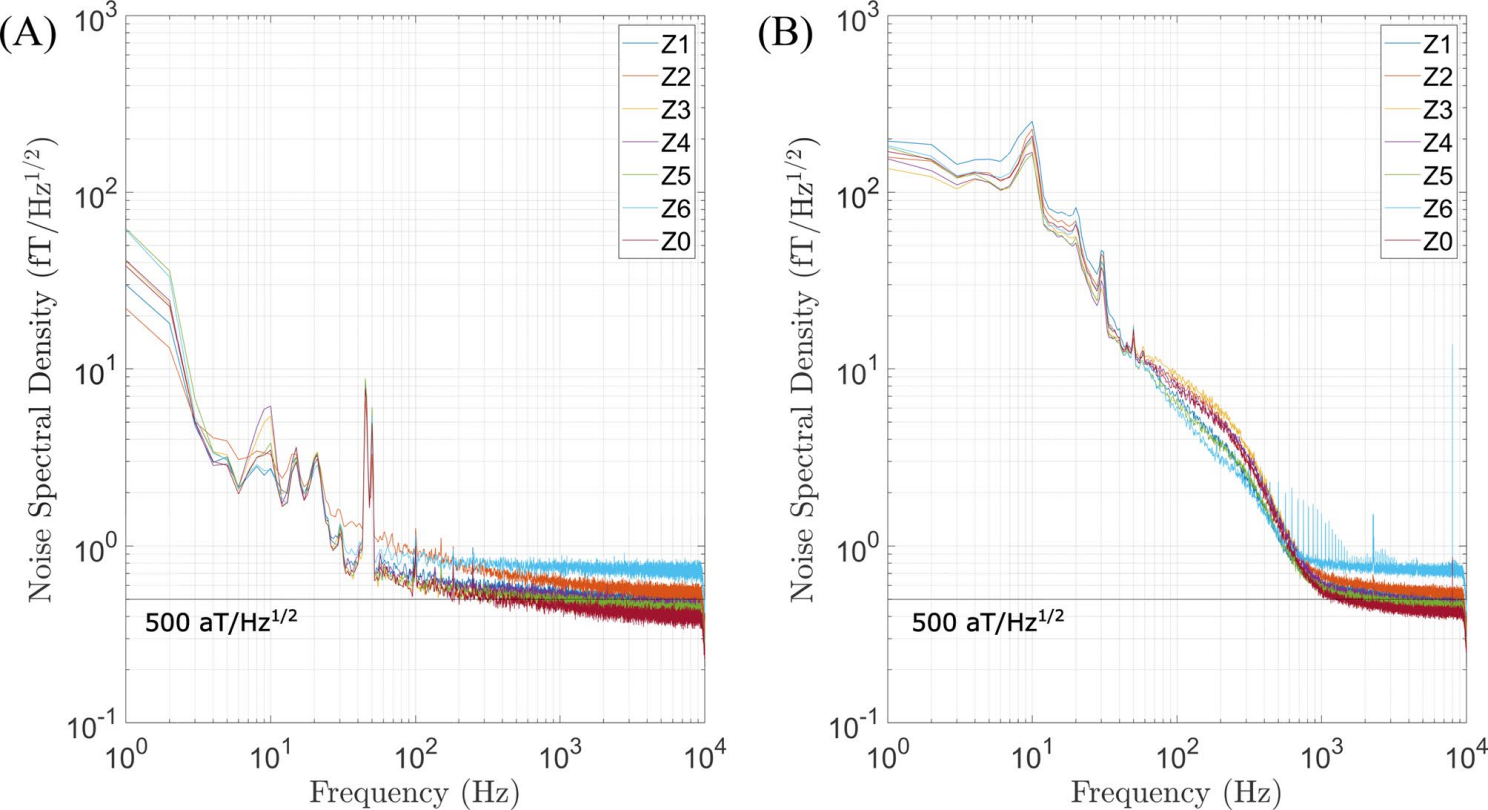
Noise spectral density of empty room (**A**) and of subject at rest (**B**) for channels Z0–Z6. The increase in noise below 1 kHz is due to physiological signals. The overall noise level is higher than expected. Software gradiometry has been implemented.

Characterisation and in vivo demonstration experiments were conducted with the 12-channel system placed centrally in the ZUSE-MSR. Figure 7 shows the noise performance for the *z*-signal channels for the empty room (A) and also a resting subject measurement (healthy 24 year-old male) (B). The application of the electronic SQUID reset procedure was not required in this session. The empty room noise level in the $\sigma$-burst range of about $\mathrm {500~aT/Hz^{1/2}}$ is moderately higher than expected. This is due to noise contributions from 5 m wiring when operating the read-out electronics outside the MSR, preventing a noise performance of $\mathrm {\approx 380~aT/Hz^{1/2}}$ in the $\sigma$-burst frequency range. For the resting state measurements, physiological activity below about 1 kHz starts to dominate with a clear $\alpha$-wave activity peak at around 10 Hz.

**Fig. 8:**
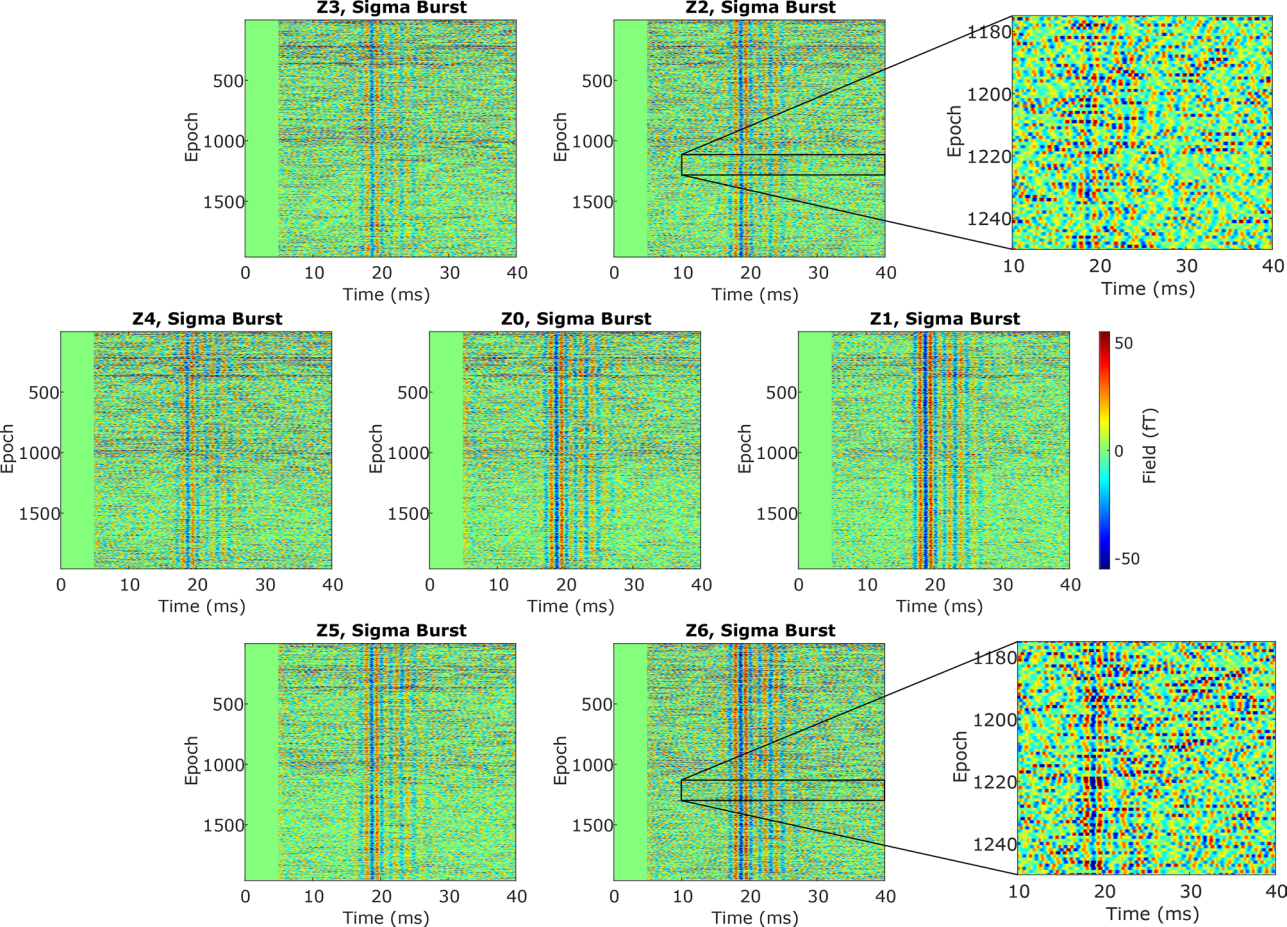
Stacked responses of channels Z0–Z6 with magnified responses of channels Z2 and Z6, to median nerve stimulation (bandpass filtered 450–850 Hz). The colour bar by Z1 applies to each plot. Each channel is orientated as seen from a top view. The $\sigma$-burst can be seen best in channel Z1. This channel is closest to a maximum of the response with the remaining channels on the same lobe of the dipolar field.

In Fig. 8, the subject’s high frequency responses following median nerve stimulation are shown for the channels Z0-Z6. Here, we applied a digital bandpass from 450 to 850 Hz and software gradiometry using the reference channel Z7 (200 $\mu$s long stimulation current of 2.5 mA and frequency of 3.27 Hz, recording time 10 minutes). The epochs are stacked on top of each other and the individual bursts can clearly be seen at the single-trial level even with a reduced empty room noise performance of $\mathrm {\approx 500~aT/Hz^{1/2}}$ in the bandwidth of interest. Due to the placement of the sensor system, the inversion of the polarity of the burst is not observed as only one lobe of the dipolar field is covered. Further detailed analysis of the results is beyond the scope of this paper, however, simple visual inspection of the magnified response (epochs 1175-1250) reveals already a different variability in the amplitude in channels Z2 and Z6, hinting to multiple underlying sources, a feature that is only possible in simultaneous multi-channel recordings. The Z1-channel for this recording gave the best signal-to-noise ratio (SNR) of 1.57. For comparison, a commercially available SQUID system with a noise of $\mathrm {2~fT/Hz^{1/2}}$ and a larger warm-cold distance of $\mathrm {\approx 28~mm}$, would have an SNR of $\approx {0.3}$, precluding single-trial detection (assuming a $\mathrm {1/r^2}$ dependence). Further studies with a larger number of subjects will exploit the capability of the ultra-sensitive 12-channel device to the full extent. Informed consent has been attained from all individuals included in this study. All methods were performed in accordance with the relevant guidelines and regulations. This research has been given ethical approval by the Ethics Commission of PTB (project ’SPIKE MEG’ (PTB2022-2)).

## Conclusions

We have described the design and realisation of an ultra-sensitive 12-channel SQUID-MEG device. The pick-up loops have been configured to have as low a field noise as possible within the limited available space of the ultra-low noise dewar, while enabling sufficient spatial resolution for recording hfSERs from different locations in the somatosensory cortex. Opting for hexagonal double-turn pick-up coils reduces the influence of stray inductance and achieves close to optimum performance. Simulations of the expected neuronal field due to the hfSERs for our sensor geometry were also performed. In addition, measures improving the dynamic range of the entire system by utilizing a SQUID reset pulse have been presented. All of these methods decrease the noise level of our device and facilitate the spatially resolved detection of the hfSERs at the single-trial level. Even though the system has been optimised for this particular neuronal response, it will also be suitable for the investigation of other cerebral cortex activations.

The multi-channel SQUID-MEG system can then be concurrently operated with a custom built low-noise 8-channel EEG amplifier that has already been used in detecting neocortical population spikes non-invasively^39^, and been shown to work simultaneously with a single-channel MEG system in the single-trial detection of hfSERs^40^. This bimodal setup enables sensitivity to both radial and tangential neuronal currents representing a leap forward in non-invasive measurements. The first MEG only recordings show the devices’ capabilities, while future work will focus on improving the device’s noise performance and data analysis from a physiological standpoint. As a final comment, we point out that further improvements are possible by replacing the current PTB current sensing SQUID. By reducing the size of the Josephson Junctions to the sub-$\mu$m range, coupled energy sensitivities as low as approximately 10 *h* and hence significant improvements can be achieved^41,42^.

## Data Availability

The data shown in this study are available from the corresponding author upon reasonable request
